# The protective effect of natural medicines in rheumatoid arthritis via inhibit angiogenesis

**DOI:** 10.3389/fphar.2024.1380098

**Published:** 2024-05-31

**Authors:** Chang Gao, Xiao-Di Song, Fang-Hui Chen, Gui-Lin Wei, Chun-Yu Guo

**Affiliations:** ^1^ Department of Pharmacy, First Affiliated Hospital of Gannan Medical University, Jiangxi, Ganzhou, China; ^2^ Gannan Medical University, Jiangxi, Ganzhou, China

**Keywords:** rheumatoid arthritis, angiogenesis, mechanisms, natural medicines, ingredients, VEGF

## Abstract

Rheumatoid arthritis is a chronic immunological disease leading to the progressive bone and joint destruction. Angiogenesis, accompanied by synovial hyperplasia and inflammation underlies joint destruction. Delaying or even blocking synovial angiogenesis has emerged as an important target of RA treatment. Natural medicines has a long history of treating RA, and numerous reports have suggested that natural medicines have a strong inhibitory activity on synovial angiogenesis, thereby improving the progression of RA. Natural medicines could regulate the following signaling pathways: HIF/VEGF/ANG, PI3K/Akt pathway, MAPKs pathway, NF-κB pathway, PPARγ pathway, JAK2/STAT3 pathway, etc., thereby inhibiting angiogenesis. Tripterygium wilfordii Hook. f. (TwHF), sinomenine, and total glucoside of Paeonia lactiflora Pall. Are currently the most representative of all natural products worthy of development and utilization. In this paper, the main factors affecting angiogenesis were discussed and different types of natural medicines that inhibit angiogenesis were systematically summarized. Their specific anti-angiogenesis mechanisms are also reviewed which aiming to provide new perspective and options for the management of RA by targeting angiogenesis.

## 1 Introduction

Rheumatoid arthritis (RA) is a chronic autoimmune disease whose main pathology is characterized by damage to articular cartilage and bone tissue, synovial hyperplasia, chronic inflammation and pannus formation (Díaz-González and Hernández-Hernández, 2023). Its worldwide prevalence ranges from 0.5% to 2% and the incidence is higher in females than in males (Radu and Bungau, 2021). RA cause progressive joint destruction, lower life expectancy, early unemployment, and considerable disability, all of which have a negative impact on patients’ health (Scherer et al., 2020; Conforti et al., 2021; Finckh et al., 2022). The pathogenesis of RA is not yet fully understood and may be related to genes, epigenetics, environmental factors (Giannini et al., 2020). Patients who have suffered irreparable damage to their joints will never regain normal physical functioning, even if clinical remission is achieved at a later stage (Frazzei et al., 2023). Early recognition and treatment can limit radioactive joint damage, preserve joint function, and achieve long-term drug-free remission (Tan and Buch, 2022). Currently, nonsteroidal anti-inflammatory drugs, antirheumatic medicines, glucocorticoids, and immunosuppressants are the most commonly used drugs in the clinical prevention and management of RA (Zhao J. et al., 2021). The first-line treatment for RA is anti-rheumatic drugs, which interferes with RA symptoms, enhance physical function, and alleviate joint deterioration. However, the progression of damage and severe incapacity is not halted by antirheumatic medicines (Lin et al., 2020). In the last three decades, new treatment alternatives have been developed for both biologics and small-molecule drugs. However, because to the complex pathophysiology of RA, it is difficult for existing drug treatments to achieve satisfactory efficacy (Smolen, 2020).

Angiogenesis, the process by which pre-existing vessels grow into new capillaries, has been implicated in the proliferation of cancers as well as the pathophysiology of inflammatory disorders (Carmeliet, 2003; Folkman, 2006). Some compounds such as nintedanib which targeting angiogenesis have already been used for tumor treatment (Roth et al., 2015). Thus, the blockade of angiogenesis was a viable strategy that could lead to remission of disease progression (Balogh et al., 2019). Angiogenesis is an early indication of RA, and is also one of the hallmarks of RA, which precedes the appearance of other symptoms (Szekanecz et al., 2009). The neovasculature generated in the initial stages of RA transports oxygen and nutrients to the highly dividing synovial tissue, increases synovial cell proliferation, and allows immune cells to invade cartilage and bone tissue (Szekanecz et al., 2005). Once the new vasculature and the proliferative synovial cells form into the vascular cartilage junction and the vascular bone junction, the invasion may injure both bone and cartilage tissue, prompting deformation of the joints and functional impairment (Maeda et al., 2022). Inhibition of neovascularization is a significant aim in the management and recovery of RA because it prevents the occurrence of pannus (Eelen et al., 2020).

Natural medicines has been used for centuries in the management of RA, which has the advantages of abundant resources, minimal toxicity and favourable therapeutic effect (Wang et al., 2021a; Liu X. et al., 2022). Some natural medicines have been offered to patients with RA or have shown encouraging evidence in preclinical studies, indicating potential for future use (Zhou et al., 2018; Liu X. et al., 2022; Luo et al., 2023). Researchers have made major advances in understanding the material basis and mechanism of natural medicines (Li W. et al., 2023). Many researches have demonstrated that natural medicines has significant anti-arthritis potential. The mechanism involved in this effect include immunological regulation, anti-oxidative stress, anti-angiogenesis, inhibition of bone destructionand and decreased expression of inflammatory indicators (Lü et al., 2015; Zhao X. et al., 2021; Liu X. et al., 2022). Firstly, the functions of angiogenesis in RA will be discussed in this review, followed by an increased overview of the angiogenic mediators required for RA. We will then describe and summarize the natural medicines including compounds, extracts, and prescription which inhibit synovial angiogenesis. Natural products primarily regulate the hypoxia-inducing factor (HIF)/vascular endothelial growth factor (VEGF)/angiotensin (Ang) signaling axes, as well as related pathways such as mitogen-activated protein kinase (MAPK), nuclear factor κB (NF-κB), protein kinase B (AKT)/mTOR, peroxisome proliferator-activated receptor-γ (PPARγ), and janus kinase/signal transduction and transcriptional activator 3 (JAK/STAT3). Relevant researches were retrieved from PubMed, Web of Science and China National Knowledge internet databases. The search algorithm was as follows: (“rheumatoid arthritis” OR “anti-rheumatic drugs”) AND (“alkaloids” OR “natural medicines” OR “flavonoids” OR “terpenoids” OR.

**FIGURE 1:**
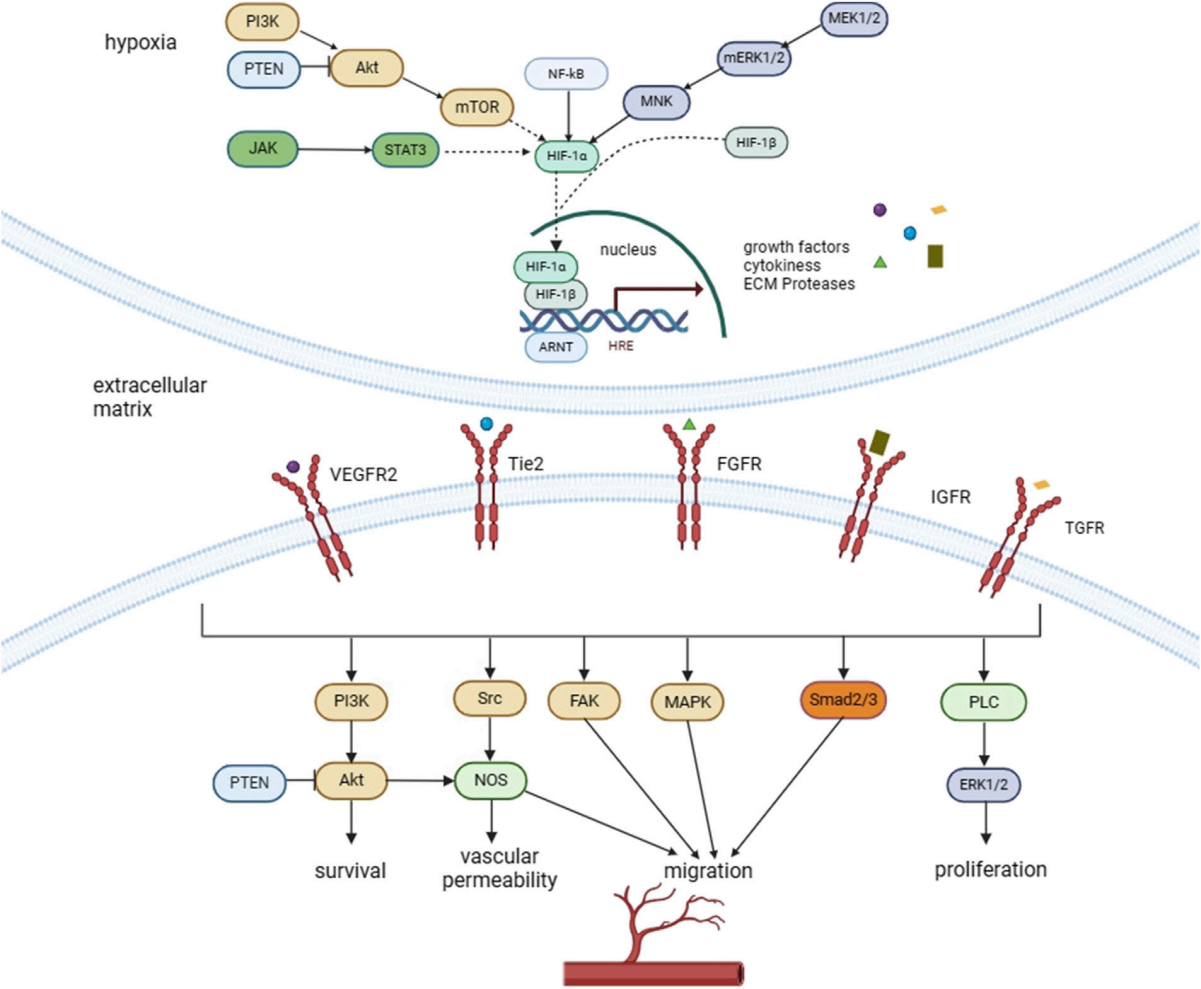
Major signaling pathways that affect angiogenesis.

“polyphenol” OR “natural extracts”). The main information were summarized, as shown in Table 1–3. The major signaling pathways that affect angiogenesis was shown in Figure 1. Studies on natural medicines that inhibit angiogenesis range from 2000 to 2023.

**TABLE 1:** Effects of compounds on angiogenesis in rheumatoid arthritis.

Categories	Natural product	Plants source	Experiment models	Effective dose	Mechanism	Ref.
Alkaloids	Sinomenine	Sinomenium acutum	CIA rats	30, 100, 300 mg/kg	HIF-1α, VEGF, Ang-1, CD31↓	Feng et al. (2019)
	Berberine	Hydrastis canadensis/Cortex phellodendri/Rhizoma coptidis	CIA rats	200 mg/kg	TNF-α, IL-1β, IL-6, IL-17, VEGF↓; VEGF, CD34↓; p-ERK, p-p38, p-JNK↓	Wang et al. (2014)
	1-Methoxycarbony-β-carboline	Picrasma quassioides	HUVECs	50 μmol/L	Ang, EGF, bFGF, GRO, IGF-1, PLG, MMP-1, TIE-2, uPAR↓	Lin et al. (2018)
			zebrafish	12.5, 25, 50 μmol/L	embryonic angiogenesis in zebrafish↓; angiogenesis in the zebrafish caudal fin regeneration assay↓	
	Matrine	Sophora flavescens Alt	CIA rats	100 mg/kg	IL-1β, IFN-γ, VEGF, PLGF, HIF-α, Ang-1, Ang-2, Tie-2↓; phosphorylation-Akt↓	Ao et al. (2022)
			FLS	0.5, 1.0, 1.5, 2.0 mg/mL	proliferation and migration of RA-FLS↓	
			HUVECs	0.5, 1.0, 1.5, 2.0 mg/mL	proliferation and lumen formation of HUVECs↓	
Flavonoids	Morin	Morus alba L	CIA rats	20, 40, 80 mg/kg	TNF-a, IL-6↓; IL-10↑; CD31, VEGF, bFGF↓	Yue et al. (2018)
			HUVECs	1, 3, 10, 30, 100 mol/L	VEGF induced migration and tube formation↓	
	Morin	Morus alba L./Otostegia persica	CIA rats	80 mg/kg	PTEN↑; PPARγ↓	Zeng et al. (2015)
			HUVECs	10 μM	migration and tube formation↓; PTEN↑, PPARγ↓; PI3K/Akt↓	
	nobiletin	citrus fruits	CIA rats	100, 400 mg/kg	IL-1β, MCP-1, IL-6, TNF-α↓; p38/NF-κB↓	Yang et al. (2017)
	Gambogic acid	Garcinia maingayi gambogic tree	AIA rats	1, 5, 10 mg/kg	IL-1β, IL-6↓; p-Akt, mTOR↑; HIF-1α, VEGF↓	Wu et al. (2017)
	Liquiritin	Glycyrrhiza uralensis	CIA rats	8 mg/kg	IL-1β, TNF-α, IL-6↓; synoviocyte apoptosis↑; VEGF↓	Zhai et al. (2019)
			RA-FLS	0.345, 3.45, 34.5 μmol/L	proliferation↓; nuclear DNA fragmentation↑; cell apoptosis↑; Bcl-2/Bax↓; VEGF, p-JNK, P38↓	
	Genistein	Euchresta japonica Benth. Ex Oliv./Sophora japonica L	CIA mice	5 mg/kg	IL-1β, IL-6, TNF-α↓; VEGF↓	Hu et al. (2016)
	apigenin	parsley/celery/celeriac/chamomile tea	CIA mice	20 mg/kg	VEGF, VEGFR↓	Li et al. (2019b)
Iridoid	Geniposide	Gardenia jasminoides Ellis	AIA mice	60 mg/kg	CD31, VEGF, p-VEGFR2, SphK1, p-SphK1, S1PR1↓	Wang et al. (2022a)
			HUVECs	25, 50, 100 μM	VEGFR2, p-VEGFR2, p-PKC, ERK1/2, p-ERK1/2, SphK1, p-SphK1, S1PR1↓	
	Geniposide	Gardenia jasminoides Ellis	AIA rats	60 mg/kg	vimentin, CD31, VEGF, VEGFR2, p-VEGFR2, Erk1/2, p-Erk1/2, SphK1, p-SphK1, S1PR1↓	Wang et al. (2021c)
			FLS/VEC	25, 50, and 100 μM	proliferation, migration, tube formation, S1P secretion↓	
	Geniposide	Gardenia jasminoides Ellis	AIA rats	30, 60 and 120 mg/kg	Dnmt1-mediated PTEN gene hypermethylation↓	Bu et al. (2022a)
			HUVECs	25, 50, 100 μM	DNA methylation, Dnmt1↓	
	Geniposide	Gardenia jasminoides Ellis	AIA rats	30, 60 and 120 mg/kg	PTEN↑; p-PI3K, p-Akt↓	Bu et al. (2022b)
			HUVECs	25, 50, 100 μM	proliferation, migration, and tubule formation↓	
	geniposide	Gardenia jasminoides Ellis	AIA rats	30, 60, 120 mg/kg	CD31, VEGF, Ang-1↓; ES↑	Sun et al. (2020)
			FLSs	5, 10, 20 μM	SphK1, S1PR1, VEGF↓	
	Geniposide	Gardenia jasminoides Ellis	AIA	30, 60, 120 mg/kg	CD31, SphK1, p-Erk1/2↓	Deng et al. (2021)
			FLSs/VECs	25,50,100 μM	SphK1, p-Erk1/2, S1P↓	
Monoterpene	Paeoniflorin-6′-O-benzene sulfonate	Paeonia lactiflora Pall	AIA rats	50 mg/kg	CXCL12, CXCR4↓	Zhang et al. (2019)
			HUVECs	1 × 10^−9^, 1 × 10^−8^, 1 × 10^−7^, 1 × 10^−6^, 1 × 10^−5^ mol/L	GRK2↓	
Sesquiterpene	artesunate	Artemisia annua L	RA FLS	0.5, 1.0, 5.0, 10, 20 μM	VEGF, IL-8↓; HIF-1α↓	He et al. (2011)
Diterpene	Triptolide	Tripterygium Wilfordii Hook f	CIA rats	11, 22, 45 mg/kg	TNF-α, IL-17, VEGF, VEGFR, Ang-1, Ang-2, Tie2↓, ERK, p38, JNK↓	Kong et al. (2013)
			HFLS–RA cells	1, 10 and 50 ng/mL	tube formation↓, chemotactic migration↓	
			HUVEC	1, 10 and 50 ng/mL	chemotactic migration↓	
Triterpene	Pristimerin	Celastrus aculeatus Merr	AIA rats	0.40,0.8 mg/kg	vessel density↓; TNF-α, Ang-1, MMP-9↓; VEGF, p-VEGFR2↓	Deng et al. (2015)
			Rat aortic ring assay	0.5, 1.0, 2.0 μM	sprouting vessels of the aortic ring↓	
			HFLS-RA	0.50, 0.75, 1.0 μM	migration↓	
			HUVECs	0.125, 0.25, 0.50 μM	VEGF-induced proliferation, migration and tube formation↓, VEGF-induced VEGFR2↓; PI3K, AKT, mTOR, ERK1/2, JNK, and p38↓	
	β-Sitosterol	many kinds of plants	CIA mice	100 mg/kg	VEGFR2, p-VEGFR2↓	Qian et al. (2021)
			HUVECs	10 and 20 μM	proliferation and migration of HUVECs↓	
Polyphenol	Chebulinic acid	Fructus Chebulae	CIA mice/DBA/1J	50 mg/kg	CD31, VEGF↓	Lu et al. (2020)
			HSMECs	1.7 μM	Erk1/2, p38 MAPK, AKT↓	
	Resveratrol	Polygonum cuspidatum	CIA rats	200, 400 mg/kg	IL-1β, MCP-1, IL-6, TNF-α, ROS↓	Yang et al. (2018)
			RSC-364	25, 50 μmol/L	HIF-1α↓; p38↓; c-Jun↓	
Anthraquinones	Emodin	Rheum palmatum L	synoviocytes	0.01, 0.1, 1, 10, 100 mM	TNF-a, IL-6, IL-8, PGE2, MMP-1, MMP-13, VEGF, COX-2, HIF-1a, HDAC1↓	Ha et al. (2011)
Coumarin	Scopoletin	Erycibe obtusifolia Benth	AIA rats	50, 100 mg/kg	blood vessel formation↓; VEGF, bFGF, IL-6↓	Pan et al. (2010)
	Scopolin	Erycibe obtusifolia Benth	AIA rats	25, 50, 100 mg/kg	IL-6, VEGF, FGF-2↓	Pan et al. (2009)
Mineral drug	arsenic trioxide	—	CIA mice	1.0, 2.0, 5.0 mg/kg	TSP-1, TGF-β1, CTGF, VEGF↓	Zhang et al. (2017)
			RA-FLS	0.5, 1, 2 μM		

↑, increase, upregulate, promote or improve; ↓, suppress, downregulate, reduce, or inhibit.

**TABLE 2:** Effects of natural medicine extracts in rheumatiod arthritis.

Phytochemicals	Animal/Strains	Dose	Effects and molecular mechanisms	Ref.
*Anemone ffaccida* Fr. Schmidt	CIA rats	200, 400 mg/kg	CD31, VEGF↓	Rao et al. (2023)
	HUVECs	5, 7.5, 10, 15, 20, 30 μg/mL	VEGFR, p-PI3K, p-AKT, p-mTOR↓	
	HFLS-RA	2.5, 5, 10, 20, 40, 80 μg/mL	Proliferation, migration↓	
*Davallia bilabiata*	chicken embryos	0.1, 0.5 mg/mL	MMP-2, MMP-14↓	Liu et al. (2017)
	HUVECs	0.25, 0.5 mg/mL^/^	VEGF-A, VEGF-B, VEGF-C, VEGF-D↓; VEGFR-1, VEGFR-2, VEGFR-3↓	
*Cissus quadrangularis*	AIA rats	50, 100, 200 mg/kg	TNF-α, IL-1β, IL-6, TNF-R1, VEGF, MMP-3, MMP-9↓	Kumar et al. (2015)
total Saponins of *Panax japonicus*	CIA mice	50, 150 mg/kg	HIF-1α, VEGF↓; SRC, STAT 3↓	Guo et al. (2020)
*Dendrobium huoshanense* stem polysaccharide	CIA mice	0.1095, 0.4380 g/kg	CD90↓; CD31↓; RANKL↓; OPG↑; VEGF, IL-17, IL-1β, IL-6, TNF-α, MMPs, GM-CSF, M-CSF, CCL5, CXCL2↓; IL-10, TGF-β1↑; NF-κB, MAPKs, PI3K/AKT, JAK1/STAT3↓; Treg cell↑; Th17 cell↓	Shang et al. (2021)
evening primrose oil	AIA rats	5 g/kg	Ang-1, TNF-a↓; SOD↓; lipid peroxidation↑	El-Sayed et al. (2014)
*Rhus verniciflua* Stokes	CIA mice	50 mg/kg	synovial inflammatory cells↓	Lee et al. (2009)
	FLS	0–1,000 μg/mL	TNF-α, IL-6, IL-8, MCP-1, VEGF↓; ERK 1/2, p-JNK, p38 MAPK↓	
saponins from *Nigella glandulifera* seeds	CIA rats	10, 50, 250 mg/kg	IFN-γ, TNF-α, IL-1β, IL-6, IL-17A↓; IL-4, IL-10↑; CD4^+^CD25^+^ Tregs↑; Foxp3↑; OPG/RANKL ratio↑; p-p65↑	Jiang et al. (2022)
	HFLS-RAs	10, 30, 100 μg/mL	proliferation, migration, adhesion↓; f TNF-α, IL-17A, Ang-2, Tie-2↓	
	HUVECs	10, 30, 100 μg/mL	proliferation, migration, adhesion↓; Ang-2, Tie-2↓	
total saponins of Rhizoma *Dioscorea* *nipponica*	CIA rats	25 mg/kg	VEGF, Ang-2, Tie-2↓; MVD, VEGF, STAT3, NF-κB↓	Liang et al. (2016)

↑, increase, upregulate, promote or improve; ↓, suppress, downregulate, reduce, or inhibit.

**TABLE 3:** Effects of natural medicine prescriptions in rheumatoid arthritis.

Prescription	Composition	Models	Dose	Effects and molecular mechanisms	Toxicity	Clinical dosage	Ref.
YuXueBi tablet	*Boswellia carteri* Birdw., *Commiphora myrrha* (Nees) Engl., *Clematis chinensis* Osbeck, *Cyathula officinalis* Kuan, *Curcuma longa* L., *Carthamus tinctorius* L., *Salvia miltiorrhiza* Bunge, *Cyperus rotundus* L., *Ligusticum chuanxiong* Hort, *Astragalus membranaceus* (Fisch.) Bunge, and *Angelica sinensis* (Oliv.) Diels	CIA rats	0.1, 0.2, 0.4 g/kg	CD31, VEGF↓; LOX, Ras, Raf-1, p-MEK p-ERK↓	eye edema, Sore throat	2.5 g p.o tid	Su et al. (2022)
		HUVECs	12.5, 25, 50, 100 μg/mL	migration, invasion, tube formation↓; LOX, Ras, Raf-1, p-MEK, MEK, p-ERK, MEK↓			
Huatan Tongluo decoction	Dannanxing (*Rhizoma Arisaematis Cum Bile*), Taoren (*Semen Persicae*), Jiangcan (*Bombyx Batryticatus*), Baijiezi (*Semen Sinapis*) and Shancigu (Pseudobulbus Cremastrae)	CIA rats	7.5 g/kg	CD34, VEGF, VEGFR2↓	gastrointestinal discomfort, dizziness	Potion p.o bid	Chen et al. (2019a)
Shexiang Zhuifeng analgesic plaster	Artificial Musk, *Aconitum kusnezoffii* Reichb., *Aconitum carmichaeli* Debeaux, *Boswellia carterii* Birdw., *Commiphora myrrha* (T.Nees) Engl., *Strychnos nux*-vomica L., *Eugenia caryophyllata* Thunb., *Cinnamomum cassia* (L.) J.Presl, *Schizonepeta tenuifolia* (Benth.) Briq., *Saposhnikovia divaricate* (Trucz.) Schischk., *Geranium wilfordii* Maxim., *Periploca sepium* Bunge, *Centella asiatica* (L.) Urban, *Drynaria fortunei* (Kunze ex Mett.) J.Sm., *Angelica dahurica* (Hoffm.) Benth. and Hook.f. Ex Franch. and Sav., *Kaempferia galanga* L., *Zingiber officinale* Roscoe, Camphor, Borneol, Menthol, Methyl salicylate, *Liquidambar formosana* Hance, *Atropa belladonna* L	CIA rats	0.63, 2.52 cm^2^	IL6, VEGF, TNF-α↓; HIF-1, p-AKT, p-mTOR↓	rash, pruritus, anaphylactic shock	External use 1 patch qd	Wang et al. (2023)
		RAW 264.7 cells	12.5, 25, 50 μM	NO↓			
Kunxian Capsule	*Tripterygium wilfordii* Hook. F. (*Tripterygium hypoglaucum* (H. Lév.) Hutch), *Epimedium brevicornu* Maxim, *Lycium barbarum* L. and *Cuscuta chinensis* Lam	Zebra fish	3.5, 7, 14, 21 μg/mL	PI3K, AKT, MAPK, ERK1, ERK2, VEGF2, VEGFR, FGF-2↓	gastrointestinal discomfort, abnormal liver function, leukopenia, paramenia, oligospermia	0.6 g p.o tid	Ma et al. (2023)
Sidaxue	*Spatholobus suberectus* Dunn, *Sargentodoxa cuneata* (Oliv.) Rehd. Et Wils., *Toddalia asiatica* L. Lam., *Periploca forrestii* Schltr	CIA rats	10, 20, 40 g/kg	IL-2, IL-6, TNF-α, VEGF, PI3K, AKT, p-AKT, NF-κBp65, p-NF-κBp65, STAT1, PTGS2↓	-	-	Wu et al. (2022)
Qianghuo Shengshi Decoction	*Ramulus Cinnamomi*, *Epimedium brevicornum* Maxim, Rhizoma Wenyujin Concisum, *Poria cocos*, *Scutellaria baicalensi*	AIA rats		IL-6, MMP-9, TNF-α, VEGFA↓	gastrointestinal discomfort, abnormal liver function, rash, infect, subcutaneous hemorrhage	Potion p.o bid	Zeng et al. (2021b)
Fengshi Gutong capsule	*Aconiti Radix Cocta*, Aconiti Kusnezoffii Radix Cocta, *Carthami Flos*, *Chaenomelis fructus*, *Mume Fructus*, Ephedrae Herba, *Glycyrrhizae Radix* et Rhizoma	CIA rats	300, 900 mg/kg	RF, VEGF, TNF-α, IL-6↓; ICAM-1, IL-1β, p-Akt↓	gastrointestinal discomfort, renal damage	0.6–1.2 g p.o bid	Lin et al. (2021)
		RAW264.7	Different concentration	IL-1β, iNOS↓			
		HUVEC	Different concentration	migration↓			
Qing-Luo-Yin Extract	*Sophora flavescens* Ait., *Phellodendron amurense* Rupr., *Sinomenium acutum* Rehd. Et Wils. And *Dioscorea hypoglauca* Palib	CIA rats	0.3 g/kg	MMP-3↓, TIMP-1↑	-	Potion p.o bid	Li et al. (2003)
Wen Luo Yin	Radix Aconiti Lateralis Preparata, Cinnamomi Ramulus, Atractylodis Macrocephalae Rhizoma, Selaginellae Herba	CIA rats	3.45, 6.9, 13.8 g/kg	TNF-α, IL-1β, VEGF↓	-	Potion p.o tid	Liu et al. (2013)
		HFLS-RA	8, 16, 32 mg/mL	migration, adhesion↓; TNF-α, IL-17, VEGF, Ang-1, Ang-2↓			
		HUVEC	8, 16, 32 mg/mL	migration, adhesion↓; VEGFR, Ang-2↓			
Wutou Decoction	*Aconitum carmichaeli* Debeaux., *Ephedra sinica* Stapf, *Paeonia lactiflora* Pall., *Astragalus mongholicus* Bunge, *Glycyrrhiza uralensis* Fisch. ex DC.	CIA rats	3.75, 7.5 g/kg/day	CD31, VEGF, Ang-1,VEGFR2, TEK, PI3K, AKT, mTOR, HIF-1α↓	gastrointestinal discomfort	Potion p.o tid	Ba et al. (2021)
		FLS	1, 10 mg/mL	proliferation, migration, invasion↓; apoptosis↑; VEGF, Ang-1↓			
		HUVEC	1, 10 mg/mL	proliferation, migration, invasion, tube formation↓			
Wutou decoction	*Aconitum carmichaeli* Debeaux., *Ephedra sinica* Stapf, *Paeonia lactiflora* Pall., *Astragalus mongholicus* Bunge, *Glycyrrhiza uralensis* Fisch. ex DC.	CIA rats	0.95, 1.9, 3.8 g/kg	CD31↓; VEGF, VEGFR2, IL-1β, IL-17, TGF-β, PDGF, PIGF, Ang I, Ang II↓	gastrointestinal discomfort	Potion p.o tid	He et al. (2018)
		FLS	0.2, 1, 5 μg/mL	IL-1β, IL-17, VEGF, TGF-β, PDGF, PIGF, Ang I, Ang II↓			
		HUVECs	0.2, 1, 5 μg/mL	migration, invasion, tube formation↓; VEGFR2↓; AKT, ERK1/2, JNK, p38↓			

↑, increase, upregulate, promote or improve; ↓, suppress, downregulate, reduce, or inhibit.

## 2 Role of angiogenesis in RA

Angiogenesis is the generation of new branches of blood vessels from pre-existing blood vessels, requiring the degeneration of the basement membrane, stimulation, expansion, and migrating of vascular endothelial cells (ECs), and neovascularization (Folkman, 2006). Synovial cells, like usual cells, require an exchange of vital nutrients and oxygen through blood vessels in addition to the elimination of metabolic waste (Taylor and Sivakumar, 2005). Under normal physiological conditions, the vascular system will be in a resting state for a long time, and only when the body is damaged will there be temporary angiogenesis (Maruotti et al., 2006).

**FIGURE 2:**
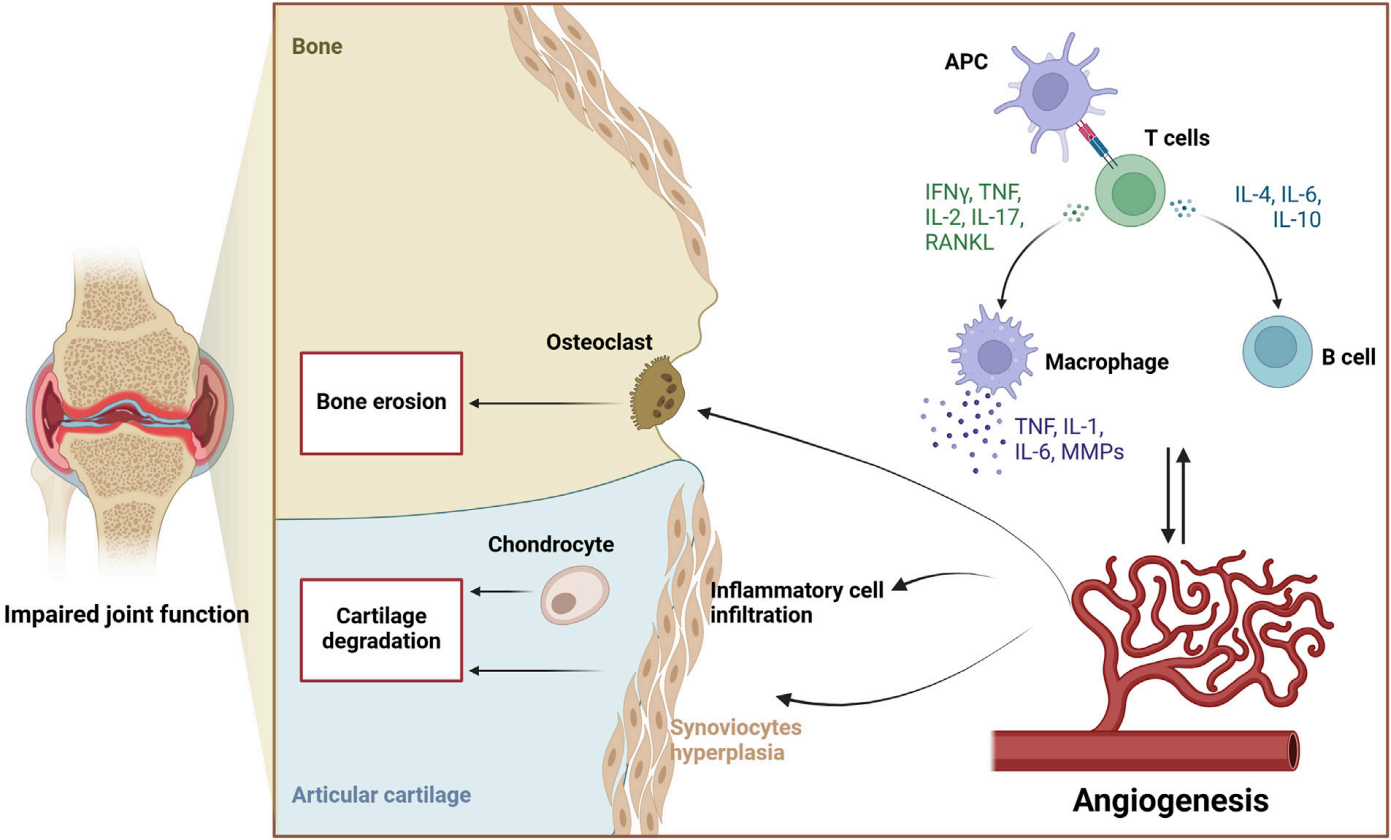
The role of angiogenesis in the pathological process of rheumatoid arthritis.

However, in the pathology of RA, the oxygen and nutrient requirements of the synovium incrementally increase, and the proliferation and expansion of synovial tissues force the number and density of synovial vessels to increase compensatively (Leblond et al., 2017). The higher the degree of synovial cell growth and the degree of monocyte infiltration, the higher the vascular density and the degree of ECs growth (Taylor and Sivakumar, 2005). Angiogenesis depends on multiple phases, each of which is modulated by particular factors. Angiogenesis is supposed to begin with the stimulation of ECs mainly activated by pro-angiogenic substances especially VEGF. The activated ECs secrete matrix metalloproteinases (MMPs) which fragment the basal membrane and trigger invasion. This causes local blood vessels dilatation, increased vascular permeability, and proteolytic breakdown of the basal membranes of existing capillary endothelial cells. When the basement membrane is disrupted, endothelial tip cells will protrude and migrate approach the site of origin of the angiogenic signaling. Following EC growth, capillary buds form, causing ECs to elongate and organize properly. The expanding buds eventually generate lumens, allowing these tubular structures to connect to nearby blood vessels. In the last stage, pro-angiogenic factors such as Ang1 stabilise blood vessels, and then pericytes are incorporated into the newly constructed basement membrane to facilitate the process of blood flow (Taylor and Sivakumar, 2005; Szekanecz et al., 2010; Rizzi et al., 2017; Eelen et al., 2020). Early neovascularization in RA strengthens tumor-like synovial cell proliferation and allows inflammatory cells to infiltrate cartilage and bone tissue. Inflammation, immunological imbalance, and angiogenesis are all linked together to promote pannus production, which damages joints and causes abnormalities in joints and dysfunction (Costa et al., 2007; Leblond et al., 2017). Numerous investigations have indicated that angiogenesis occurs at the beginning of RA and persists throughout every phase of the disease. There is evidence that the suppressing angiogenesis may improve synovial inflammation and pannus formation. Consequently, angiogenesis is essential to both the initiation of RA and the discovery of effective pharmaceuticals (Semerano et al., 2011; Wang et al., 2021b). The role of angiogenesis in the pathological process of rheumatoid arthritis is shown in Figure 2.

## 3 The major mediator and signaling pathways that regulates angiogenesis

Angiogenesis is mainly regulated by an appropriate ratio of promoting and inhibitory forces (Eelen et al., 2020). Once this balance is broken, it will activate the angiogenesis into new vessels or suppress the vasculature angiosclerosis. Synovial neovascularization can be aided by a variety of mediators, such as growth factors, cytokines, chemokines and so on. Conversely, synovial angiogenesis is hampered by endogenous vascular inhibitors, including angiostatin, interleukin-4 (IL-4), interleukin-13 (IL-13) and interferon. Among them, VEGF, HIF-1, Ang-1, Ang-2 are recognized as most important angiogenic factors that affect synovial angiogenesis (Szekanecz et al., 2005; Maruotti et al., 2006).

### 3.1 VEGF

VEGF is a homologous dimeric glycoprotein which involved in regulating neovascularization. The VEGF family is a group of angiogenic cytokines that comprises placental growth factor (PLGF), VEGF-A→VEGF-F (Costache et al., 2015; Hamilton et al., 2016). The importance of VEGF-A (commonly referred to as VEGF) in the regulation of angiogenesis will be the primary topic of this review (Apte et al., 2019). VEGF experience alternative exon splicing, resulting in various isoforms. Among them, VEGF165 is the most widely expressed isoform in tissues (Woolard et al., 2009). VEGF has two high-affinity receptors, VEGFR-1 and VEGFR-2, which are predominantly distributed on ECs, and a small amount of other cells such as hematopoietic stem cells and monocytes are also expressed, but it is currently believed that only ECs have a response to VEGF (Melincovici et al., 2018). Studies have shown that VEGF specifically acts on vascular ECs and can affect their proliferation, differentiation and other functions. The VEGF-dependent signaling pathway plays a central part in angiogenesis during RA pathophysiology (Liang et al., 2014). Under normal conditions, vascular ECs renewal is slow and VEGF expression is low. During tissue injury and inflammation, hypoxia, platelet-derived growth factor (PDGF), fibroblast growth factors (FGFs), etc., Can increase VEGF by 3–20 times (Melincovici et al., 2018). VEGF signaling modulates the activation of various kinases during vasculogenesis and angiogenesis, which in turn controls cell division, movement, longevity, and permeability of the vessel (Ahmad and Nawaz, 2022).

### 3.2 Ang

Ang serves as one of the growth factors requested for the initial angiogenesis. At the present time, the Ang family is the sole known pro-angiogenic factor having both promoting and inhibitory actions, and it consists four members, Ang-1→Ang-4, which all share the EC tyrosine kinase receptor Tie-2 (Saharinen et al., 2017). Ang-1 and Ang-2 are two key angiogenin regulators involved in vascular development, endothelial eruption, vascular wall remodeling and parietal cell recruitment. According to research, the Ang/Tie2 signaling pathway is directly associated to the development of blood vessels in pathological diseases such as RA and tumor (Fagiani and Christofori, 2013). There is 60% similarity between Ang1 and Ang2, in addition, they all have high binding affinity with Tie2. Ang-1 adheres to and phosphates the Tie2 receptor, which stimulates it and facilitates the survival of cells and vascular integrity. Ang-1/Tie2 signalling also inhibits NF-κB pathway thus decreasing the inflammatory factor cascade and maintaining vascular stability (Mitola et al., 2008). Overall, the Ang1/Tie2 signaling pathway builds vascular stability and quiescence under physical environments (Fukuhara et al., 2010). In pathological conditions, Ang-2 expression is increased, which pushes the emergence of RA’s clinical hallmarks. As natural antagonist of Ang-1, Ang-2 is employed to cause EC instability and prevent Ang-1-induced Tie2 phosphorylation. In fact, the Ang/Tie signal pathway and the VEGF/VEGFR signaling pathway are related to each other and coordinate with each other to regulate the processes of vascular growth, maturation and degeneration (Akwii et al., 2019).

### 3.3 HIF-1

Hypoxia predominantly modulates VEGF expression by means of HIF, a heterodimeric transcription factor with α (HIF-1α) and β (HIF-1β) subunits (Hamilton et al., 2016; Yang et al., 2021). Under proper condition of oxygen, HIF-1α expression is limited. Hypoxia causes HIF-1α to aggregate and bond to HIF-1β, generating a stable active dimer HIF-1 (Balamurugan, 2016). HIF-1 encourages synovial angiogenesis by prompting synoviocytes to release VEGF, which influences the progression of RA. Additionally, synovial angiogenesis boosted HIF-1α and VEGF amounts of expression in synovial tissues (Westra et al., 2010; Konisti et al., 2012).

### 3.4 Growth factors

Growth factors including FGF, PDGF, transforming growth factor-β (TGF-β), insulin-like growth factor 1 (IGF-I), hepatocyte growth factor (HGF) and epidermal growth factor (EGF) also promote angiogenesis (Szekanecz and Koch, 2009). FGF is secreted by ECs in the pannus and includes alkaline FGF and acidic FGF. The former (bFGF) can stimulate microvascular ECs to secrete metalloproteinases and destroy vascular endothelial matrix. aFGF not only produces collagenase and plasminogen, which degrades ECs’ basement membranes, but it also has a potent mitogenic impact and directly induces the proliferation of vascular ECs. (Malemud, 2007). PDGF and TGF-β both helps blood vessels function properly (Carmeliet and Jain, 2011). PDGF is needed for the recruitment of pericytes to newly established vessels. During angiogenesis, sprouting ECs secrete PDGF which stimulates proliferation and migration of mural cells during vessel maturation (Armulik et al., 2005). The reinduction of vascular smooth muscle cells around neovasculature is dependent on TGF-β, which is required for the growth and function of the vasculature (Armulik et al., 2005).

### 3.5 Pro-inflammatory cytokines

Cytokines leave their mark throughout the whole course of RA’ biological development (Kondo et al., 2021). Pro-inflammatory cytokines including TNF-α, IL-1, IL-6, IL-15, IL-17, IL-18 promote synovial vessel formation (Szekanecz and Koch, 2009). Studies on the effects of TNF-α and IL-6 on angiogenesis are the most in-depth and extensive (Kondo et al., 2021). Tumor necrosis factor (TNF-α) may stimulate a multitude of immune cells in the RA cartilage tissues to produce pro-angiogenic factors, which in consequently results in persistent inflammation of the synovial tissue during the progression of the disease (Chen Z. et al., 2019; Kondo et al., 2021). IL-6 is produced by monocytes and macrophages of RA synovium, which can activate vascular ECs(Zegeye et al., 2020). It not only boosts pannus formation but also aggravates bone resorption (Pandolfi et al., 2020). Anti-inflammatory cytokines, such as interferon-α (IFN-α), IFN-γ, IL-4, IL-12, IL-13 and leukemia inhibitory factor (LIF) are mediators that inhibit angiogenesis (Szekanecz and Koch, 2009).

### 3.6 Others

Angiogenesis regulation is a complex process. Except the above-mentioned factors, there are also chemokines/receptors, extracellular adhesion molecules and proteolytic enzyme possess a contribution to make in the controlling of synovial angiogenesis (Maruotti et al., 2006). Matrix metalloproteinases (MMPs) and plasminogen activators participate in the dissolution of synovial cartilage during the maintenance of angiogenesis. Other factors that both encourage and inhibit angiogenesis that are mentioned have been reviewed in the literature (Paleolog, 2002; Balogh et al., 2019). Plenty of studies on RA have proved that extracellular adhesion molecules play a critical part in leukocyte migration into synovial tissue (Volin, 2005).

### 3.7 Signaling pathways

In addition, various additional signaling pathways are also associated in angiogenesis. MAPK, NF-κB, JAK-STAT, PI3K, and PPARγ signaling pathways have been extensively studied for their role in inducing angiogenesis. The PPARγ signaling pathway is capable of promoting angiogenesis by enhancing VEGF expression. Signaling pathways such as MAPK, NF-κB, and JAK/STAT3 may indirectly boost angiogenesis. MAPK mediates various cellular physiological processes and abnormal activation of MAPK signaling pathway can lead to RA. MAPK signaling pathway is a tertiary enzyme linked system with four activation pathways: ERK1/2, JNK/SAPK, p38 and ERK5. In the pathological process of RA, MAPKs not only regulate the production of pro-inflammatory cytokines and matrix protein degrading enzyme, but also play an important role in the downstream signaling cascade of cytokine receptors (Liu et al., 2021). MAPK is the up-stream signal of the NF-κB pathway and is regarded as an intersection of various signalling pathways (Yeung et al., 2018). NF-κB is an important transcription factor in the cytoplasm. The expression of a large number of genes in immune and inflammatory responses is regulated by NF-κB. The role of NF-κB in the pathogenesis of RA has attracted the attention of researches. Activation of NF-κB signaling pathway can lead to T cell activation to induce the production of various pro-inflammatory cytokines. It can also induce abnormal proliferation of RA-FLS to stimulate osteoclast proliferation and activation, which results in joint deformity and bone erosion (Liu et al., 2021). After activation of JAK-STAT pathway, it can activate the activity of immune cells, especially T cells, and inhibit FLS autophagy, resulting in continuous proliferation of RA synovium and cartilage erosion (Di Benedetto et al., 2021). PI3K pathways have a key role in angiogenesis which involved in stimulating HIF(Zeng et al., 2016). These multiple pathways interact and collaborate to control diverse angiogenic activities in vascular cells. Animal model for rheumatoid arthritis commonly used are shown in Table 4.

**TABLE 4:** Animal model for rheumatoid arthritis commonly used.

	name	Molding method	Suitable species	Molding time	Position
induced model	collagen-induced arthritis (CIA)	CⅡ + CFA	rat, mice, monkey	21–25 days	base of the tail, dorsal skin
	collagen-antibody-induced arthritis (CAIA)	CⅡ + LPS	mice	7 days	base of the tail
	adjuvant-induced arthritis (AIA)	CFA + tubercle *bacillus*	rat	10–14 days	base of the tail, hind paw region
	streptococcal cell wall induced arthritis (SCWIA)	PG-PS	rat, mice	3 days	intra-articular
	COMP induced arthritis	IFA + COMP	rat, mice	45 days	base of the tail
	pristane induced arthritis (PIA)	2,6,10,14-tetramethylpentodecane	rat, mice	2 months	intradermal injection
	antigen induced arthritis	mBSA + CFA	mice, rats, guinea pigs, rabbits	6 days	subcutaneous or intradermal injections, intra-articular
	proteoglycan induced arthritis (PGIA)	Proteoglycan + DDA	rabbit, dogs, mice	35 days	intra-articular
	G6PI induced arthritis	G6PI + CFA	mice	15 days	base of the tail
genetic models	K/BxN mice	-	mice	10–14 days	-
	TNF-α mice	-	mice	3-4 w	-
	SKG mice	-	mice	14 days	-
	IL-1ra−/− transgenic mice	-	mice	5 w	-

## 4 Natural medicine targeting angiogenesis to prevent and treat RA

### 4.1 Natural active ingredients

**FIGURE 3:**
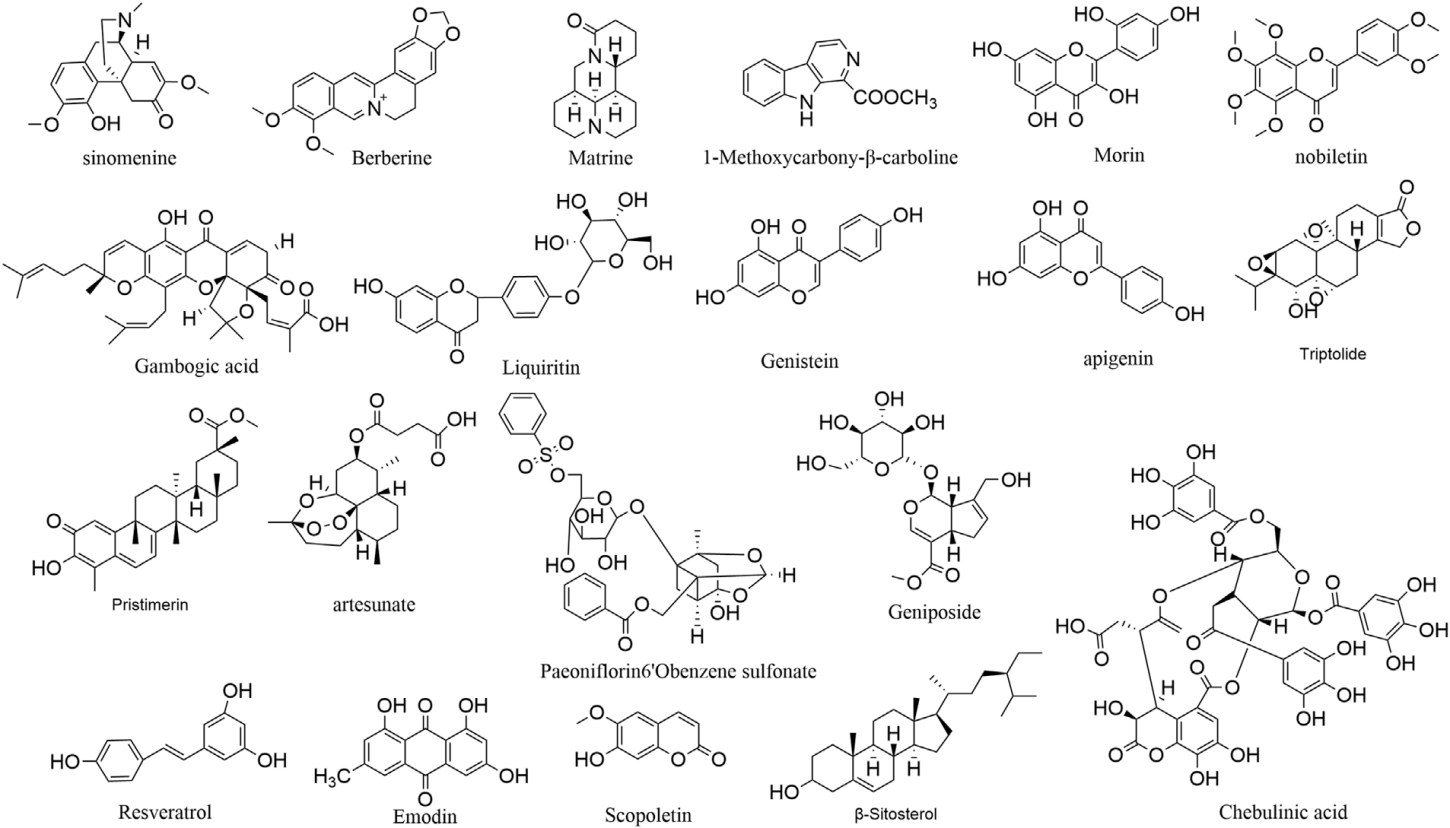
The chemical structures of compounds in natural medicine with antiangiogenesis effects.

Natural medicines contain abundant anti-angiogenic active ingredients, including alkaloids, flavonoids, terpenoids, polyphenol and so on. Figure 3 illustrates the chemical structures of the active components obtained from natural medicines. Table 1 summarizes the models and mechanisms of all these components against rheumatoid arthritis.

#### 4.1.1 Alkaloids

Alkaloids occur naturally as organic molecules with no less than one basic nitrogen atom embedded in their ring structure. Alkaloids can be divided into organic amines, pyrrolidines, pyridines, isoquinolines, indoles, scopolanes, imidazoles, quinazolines, purines, steroids, terpenes and others according to their structure (Bhambhani et al., 2021). Alkaloids are employed in the clinical treatment of a number of illnesses. For example, berberine is used to treat enteritis that achieve significant benefits (Cao et al., 2023). Alkaloids are found in numerous natural medicines, including *tricuspidus*, *aconitum*, *ephedra*, *corydalis*, and *tetrandrhexis*. They are also extensively distributed in nature (Bhambhani et al., 2021). Many alkaloids have therapeutic effects on experimental arthritis (Lu et al., 2022), among which sinomenine has been used in clinic.

Sinomenine, the main component of *Sinomenium acutum*, has been traditionally utilized as a natural medicine for RA. Sinomenine has been proven in modern pharmacological research to be capable of remarkable anti-inflammatory, pain reliever, and immunosuppressive effects (Li J. M. et al., 2023). In collagen-induced arthritis (CIA) mice, sinomenine can significantly reduce the swelling, erythema expansion, arthritis index, cartilage deterioration, bone erosion and the quantity of CD31-positive cells in the synovium. In addition, sinomenine can drastically lower the levels of HIF-1α, VEGF and Ang-1 in the peripheral blood. The HIF-1α-VEGF-Ang-1 axis is considered to be essential for the inhibitory effect of sinomenine on angiogenesis (Feng et al., 2019). Besides sinomenine, other alkaloids also provide inhibitory effects on RA angiogenesis as well.

Berberine, a quaternary ammonium alkaloid, as an active ingredient in various therapeutic materials (such as *Hydrastis canadensis*, *Cortex phellodendri*), has a number of pharmacological actions which include antimicrobial and anti-diarrhea (Song et al., 2020). Wang et al. found that berberine considerably reduced the hyperplasia of synovial tissue and inflammatory responses in CIA rats, while also suppressing cytokines and VEGF in the plasma. Subsequent investigations into beberine’s mechanism showed that it inhibited the activation of phospho-extracellular signal-regulated kinase (p-ERK), phosphorylated p38 mitogen-activated protein kinase (p-P38), and phosphorylated c-Jun N-terminal kinase (p-JNK). Additionally, it markedly decreased the generation of VEGF and CD34 (*p* < 0.05). These discoveries indicate that berberine has both anti-inflammatory and anti-angiogenic potential, meaning that it may have an important therapeutically value in RA (Wang et al., 2014). Matrine is a prominent bioactivated alkaloid gained from *Sophora flavescens* Alt, and extensive research have corroborated its wide-ranging biological activities, including anticancer, anti-inflammatory and antiviral (Sun et al., 2022). In cellular experiments, matrine suppressed the migration and proliferation of fibroblast-like synoviocytes (FLS). Additionally, it decreased the growth and lumen formation of human umbilical vein endothelial cells (HUVECs). *In vivo*, matrine has the ability to suppress the expression of several proteins, including IL-1β, interferon gamma (IFN-γ), VEGF, PLGF, HIF-α, Ang-1, Ang-2, Tie-2, and phosphorylated -akt in ankle of CIA rats. Further investigation of the mechanism revealed that matrine inhibits angiogenesis by suppressing the PI3K/Akt signalling pathway and the HIF-VEGF-Ang axis, thereby alleviating RA symptoms (Ao et al., 2022). *Picrasma quassioides* is a natural medicine, using dried branches and leaves, rich in alkaloids, triterpenoids. *Picrasma quassioides* has anti-tumor, anti-inflammatory, antiviral and antihypertensive activities, according to modern pharmacological investigations (Lee et al., 2019). Several alkaloids with anti-angiogenic activity were discovered by zebrafish bioassay-guided screening. Following screening, 1-Methoxycarbony-β-carboline (MCC) had the highest anti-angiogenic index. Further studies confirmed that MCC inhibits HUVECs activity, migration, invasion and lumen formation, as well as downregulates several angiogenesis-ralated proteins, like Ang, epidermal growth factor (EGF), bFGF, growth-related oncogene (GRO), insulin like growth factor 1 (IGF-1), plasminogen (PLG), and matrix metallopeptidase-1 (MMP-1). In zebrafish tail fin regeneration experiments, it was observed that MCC prevented the formation of blood vessels. These findings point to MCC’s potential therapeutic utility as a powerful natural angiogenesis inhibitor for RA via numerous biological targets (Lin et al., 2018).

Collectively, most alkaloids perform as antiangiogenic drugs by inhibiting angiogenic factors, lumen formation, and proliferation of RA-FLS. The two biggest obstacles to alkaloid study are their complicated structures and insufficient bioavailability. The use of modern preparation strategies and biochemical transformation techniques can address the problem of low bioavailability (Cheng et al., 2018; Valentová, 2023). Chemical synthesis provides a way to prepare the active components of natural medicines. With the application of green chemistry, biocatalysis, and computer-aided drug design, more efficiency has been gained in the synthesis of highly complex natural compound. Many difficulties, such as many chiral centers, complex synthesis steps, easily formed isomerism, and chemical agent pollution, will be solved or considerably improved by the use of the above technology (Wang et al., 2019; Chakrabarty et al., 2021; Peña et al., 2023).

#### 4.1.2 Flavonoids

Flavonoids are a group of compounds with a C6-C3-C6 skeleton, consisting of three carbon atoms connected to two benzene rings (Dias et al., 2021). These compounds are abundant in natural medicines and are mostly bonded to sugars to the formation of glycosides. They have favorable therapeutic effects such as immune enhancement, anti-aging and antioxidant which some of them including quercetin have been applied to dietary supplements (Wen et al., 2021; Shen et al., 2022).

Morin is a kind of flavonoid that is found in a diverse range of plant sources. A series of investigations have revealed that morin has potent antioxidant and anticancer effects (Rajput et al., 2021). At the same time, multiple research have confirmed that morin has potent anti-inflammatory effects (Caselli et al., 2016). *In vitro*, Yue et al. investigated the impact of morin on HUVECs migration through wound healing and transwell experiments. Morin blocks VEGF-induced HUVEC movement and tubular formation through initiating PPARγ. Morin raised expression levels of phosphatase and tensin homolog deleted on chromosome ten (PTEN), whereas suppressing PI3K/Akt signaling. Animal experimentation have demonstrated that morin can relieve symptom of CIA rats, reduce synovial angiogenesis and upregulate synovial PTEN expression in synovial membrane. In brief, morin, a putative PPARγ agonist, attenuates angiogenesis and joint inflammation by acting on the PPARγ-PTEN-PI3K/Akt pathway (Yue et al., 2018). Zeng et al. also used the CIA rat model and observed that morin can strongly downregulate the serum pro-inflammatory factors TNF-α and IL-6, upregulate the anti-inflammatory factor interleukin-10 (IL-10) and ameliorate the pathologic damage of the joint tissue. Morin was able to drastically reduced the production of CD31, VEGF and bFGF in the synovial tissue of CIA rats, while also decreasing the serum VEGF levels. These findings indicate that morin has anti-rheumatoid potential via inhibiting synovial angiogenesis (Zeng et al., 2015).

Nobiletin is a natural constituent widely occurring in the peel of *citrus* fruits (such as Pericarpium *Citri Reticulatae*) belonging to the rutaceaceae family (Chen Y. Y. et al., 2023). It can inhibit inflammation, regulates blood lipids and act as an antioxidant (Nakajima and Ohizumi, 2019). Yang et al. employed bovine type II collagen to establish a CIA model in SD rats. Nobiletin was able to drastically lower the levels of nuclear factor kappa-B kinase α (IκBα), phosphorylated p38, p-p65, and TNF-α in CIA rats. These results point to the fact that nobiline inhibited the p38/NF-κB signaling pathway, which in turn prevented the development of RA through decreased angiogenesis and inflammation (Yang et al., 2017). Garcinia, a resin, which has a number of different pharmacological activities such as anti-tumor, detoxification and hemostasis, and its main active ingredient is garcinic acid (Jia et al., 2015; He et al., 2023). Gambogic acid has potently inhibited right paw swelling in adjuvant-induced arthritis (AIA) rats, increased the pain threshold, decreased the clinical arthritis score, and downregulated pro-inflammatory markers. *Garcinia cambogia* also relieves RA by regulating the PI3K/Akt/mTOR signaling pathway, resulting in decreased inflammation and angiogenesis. Therefore, Gambogic acid might be used as a prospective treatment in the future medical management of RA (Wu et al., 2017). Liquiritin is a natural flavonoid isolated from licorice root, which has antioxidant, antibacterial and anti-tumor effects (Qin et al., 2022). Zhai et al. reported that liquiritin greatly suppressed the proliferation of IL-1β-induced RA-FLS, enhanced nuclear DNA fragmentation, altered mitochondrial membrane potential and accelerated cell apoptosis. Liquiritin can also downtregulate the proportion of B-cell lymphoma 2/Bcl-2-associated X protein (Bcl-2/Bax), inhibit VEGF expression and c-Jun N-terminal kinase (JNK) and P38 phosphorylation. In addition, liquiritin was able to improve the rheumatism score, inflammatory infiltrations and angiogenesis in CIA rats and induce synovial tissue apoptosis. Therefore, liquiritin may improve RA by controlling inflammation, blocking MAPK signalling and inhibiting angiogenesis (Zhai et al., 2019). Genistein, which is present in many medical herbs such as *Euchresta japonica* Benth. Ex Oliv. And *Sophora japonica* L., is an isoflavone compound that possesses a series of biological effects including anti-angiogenesis and joint protection properties (Sharifi-Rad et al., 2021). Genistein is able to reduce the expression of the pro-inflammatory factors in the serum of CIA mice, inhibit bone degradation, reduce synovial inflammation, inhibit VEGF expression, and block the angiogenesis of synovial tissue (Hu et al., 2016). Apigenin widespread in warm tropical vegetables, fruits and medicinal plants (Salehi et al., 2019). The study by Li et al. has indicated that apigenin is able to inhibit the proliferation of synovial cells *in vitro*. *In vivo*, apigenin has a very protective effect on CIA mice and can prevent angiogenesis in CIA mice by blocking VEGF and VEGFR. Furthermore, apigenin may inhibit the nuclear factor-κ b ligand receptor activator/receptor activator of nuclear factor-κ b/osteoprotegerin (RANKL/RANK/OPG) signaling pathway, which may help protect bones and joints from damage. Therefore, apigenin may influence the pathogenesis of RA by inhibiting synovial hypertrophy, angiogenesis, and osteoclastogenesis, thereby ameliorating arthritis manifestations (Li Y. et al., 2019).

In conclusion, flavonoids have significant antiangiogenic effects. At the same time, it has the advantages of low toxicity and abundant resources which make it has a strong potential for RA treatment (Hughes et al., 2017).

#### 4.1.3 Terpenoids

Terpenoids, which are widely distributed in higher plants, fungi, microorganisms, insects, and marine creatures, are characterized as hydrocarbons and their derivatives based on the carbon structure of 2-methylbutadiene and 3-diene (also known as isoprene) (Bergman et al., 2019; Avila, 2020; Amirzakariya and Shakeri, 2022). Terpenoids are classified as monoterpenes, sesquiterpenes, diterpenes, disesquiterpenes, triterpenes, tetriterpenes, and polyterpenes based on the amount of isoprene units they possess. Terpenoids are the most abundant class of natural substances, and more than 40,000 compounds have been identified, accounting for 60% of all natural compounds. Terpenoids are rich in diversity and have multiple biological functions (Bergman et al., 2019). In the past few decades, specialists and scholars all over the world have paid increasing attention to the anti-arthritis properties of terpenoids and their mechanisms (Lü et al., 2015). The bioactive compounds of terpenoids with anti-angiogenic effects include triptolide (TP), geniposide, Paeoniflorin-6′-O-benzene sulfonate, artesunate, Pristimerin, etc.

TP is an epoxidized diterpene lactone and a major active ingredient of *Tripterygium wilfordii* Hook. f. (Gao et al., 2021). Numerous investigations have ascribed the anti-arthritis potential of TP to its immunosuppressive, anti-inflammatory, activation of apoptosis, cartilage protection and gene regulation (Yuan et al., 2019). According to Kong et al.’s research, TP was also reported to significantly inhibit the phosphorylation of extracellular signal-regulated kinase (ERK), p38 and JNK at the protein level and to downregulate the expression of angiogenic activators. In cellular assays, TP inhibited HUVEC luminal formation and chemotactic migration of RA-FLS and ECs. The above experiments provided evidence that TP may be able to inhibit angiogenesis and is an excellent candidate as a novel therapeutic drug for RA (Kong et al., 2013). Pristimerin is a naturally occurring quinonemethide triterpenoidis which contain in *Celastrus aculeatus* Merr. (Wang et al., 2022a). In past years, more and more research has been undertaken on the anti-arthritis pharmacological effect of Pristimerin (Tong et al., 2014; Lv et al., 2022). In AIA rats, Pristimerin has been shown to significantly decrease the density of synovial vessels in inflamed joints and serum pro-angiogenic factors such as matrix metallopeptidase-9 (MMP-9). Pristimerin has also been observed to decrease the expression of VEGF and its receptor in the synovium. *In vitro*, pristimerin inhibited rat aortic ring vessel sprouting and HFLS-RA and HUVEC migrations. Pristimerin also inhibited VEGF-induced HUVEC proliferation and luminal formation and downregulated the levels of activated PI3K, AKT, mTOR, ERK1/2, JNK and p38 (Deng et al., 2015). A sesquiterpene lactone isolated from *Artemisia annua* L., artemisinin is a useful medication for the treatment of malaria (Ma et al., 2020). Artesunate is derived from artemisinin, which is used mainly as an antimalarial drug (Zhang J. et al., 2022). Artesunate may not only modulate T and B cell immunological function and limit the generation of autoantibodies, but it could also directly alter FLS and osteoclasts, inhibit synovitis and osteoclastogenesis, and therefore suppress joint inflammation and bone degradation associated with RA. As a result, artesunate may be a viable treatment option for RA in the clinic. Artesunate suppresses the production of VEGF and IL-8 as well as the nuclear expression and translocation of HIF-1α in RA-FLS, which indicates that it could inhibit angiogenesis through those pathways (He et al., 2011). Paeoniflorin, which has both an antiinflammatory and immunomodulatory function, is one of the principle active constituents of genus *Paeonia*. But its oral bioavailability is low, about 3%–4% (Zhang X. X. et al., 2022). Paeoniflorin six-o’-benzenesulphonate (CP-25) is a lipophilic molecule obtained by structural modification of paeoniflorin (Tu et al., 2019). CP-25 is a remarkable inhibitor of pannus formation in AIA rats. The specific mechanism could be associated to the lowered the plasma membrane distribution of G protein-coupled receptor kinase 2 (GRK2) in ECs, the more marked suppression of ERK1/2 in the cytoplasm by GRK2, the decreased phosphorylation of ERK1/2 (Zhang et al., 2019). Sterol is an abundant phytosterol and Qian et al. recently showed that β-sitosterol inhibits angiogenesis both *in vitro* and *in vivo*. In the cellular experiments, β-sitosterol suppressed the HUVECs’ growth. Notably, in CIA mice, treatment with β-sitosterol was more effective than axitinib in inhibiting VEGFR2/p-VEGFR2 (Qian et al., 2021).

Geniposide is iridoid glucoside, which easily soluble in water, and is the main pharmacodynamic component of *Gardenia jasminoides* Ellis. Geniposide has a considerable impact on illnesses of digestive, circulatory, and neurological systems. Additionally, Geniposide has anti-inflammatory and soft tissue injury healing properties (Liu L. et al., 2022). Geniposide treats RA by inhibiting angiogenesis through multiple pathways. Geniposide can block angiogenesis, rebalance pro/anti-angiogenic factors and suppress activation of VEGF and S1P signalling pathways in the synovium in the AIA rat model, according to the study by Wang et al. Geniposide decreases VEGFR2/PKC/ERK1/2-mediated sphingosine kinase 1 (SphK1) translocation, prevents activation of sphingosine 1-phosphate/sphingosine 1-phosphate receptor 1 (S1P/S1PR1) signalling, thereby limiting VEGF-induced angiogenesis (Wang et al., 2022b). Wang et al. also investigated the anti-angiogenic potential of geniposide, the main therapeutic targets of which are to lower the expression of VEGF, to reestablish the dynamic balance between pro- and anti-angiogenic factors, to block the SphK1/S1P signalling pathway and to decrease the secretion of S1P(Wang et al., 2021c). Bu et al. figured out that geniposide improved the severity of inflammation and angiogenesis in AIA rats. Geniposide has an anti-angiogenesis effect *in vitro* via decreased HUVEC migration, proliferation, and tubule formation. According to the mechanistic study, geniposide blocked the activation of the PI3K-Akt signalling pathway, upregulated the expression of PTEN and indirectly inhibited angiogenesis (Bu et al., 2022b; a). In experimental arthritis, geniposide confirmed anti-angiogenesis actions by preventing Dnmt1-mediated hypermethylation of the PTEN gene (Bu et al., 2022b). Sun et al. discovered that geniposide dose-dependently reduced inflammatory manifestation and synovial microvessel density (MVD) in AIA rats. Geniposide can lower VEGF and Ang-1 production, increase ES secretion, and inhibit abnormal FLS proliferation (Sun et al., 2020). Deng et al. discovered that geniposide decreased S1P secretion and the interaction between FLSs and ECs via decreased expression of p-Erk1/2 and SphK1. These findings provided insight into the process of angiogenesis in the inhibition of geniposide. These data confirm that Geniposide is able to inhibit angiogenesis through several pathways and is expected to be developed as an angiogenesis inhibitor (Deng et al., 2021).

Although terpenoids have a relatively significant effect in anti-arthritis, some of them may cause serious liver and kidney damage when used, which is not to be ignored. For instance, TP is a pharmacologically active ingredient of Tripterygium wilfordii, but its clinical application is limited due to a restricted therapy window and multi-organ toxicity. Triptolide can disrupt a number of cellular structures and functions, including membrane injury, mitochondrial disruption, metabolic malfunction (Xi et al., 2017; Cui et al., 2023). Therefore, reducing the toxicity of terpenoids and increasing their bioavailability through structural modification are very important for further application of terpenoids.

#### 4.1.4 Polyphenol

Polyphenols, as natural metabolites widely found in natural medicines, have a high variety of biological capacities such as anti-oxidation, anti-inflammation, anti-fibrosis and anti-tumor (Luca et al., 2020). Additionally, they have low toxicity and will not accumulation in body, which are potential active ingredients of great development value for dietary supplement and drugs (Gamage et al., 2023).

Chebulinic acid, obtained from *Fructus Chebulae*, is a polyphenol which has also been described to reduce inflammatory manifestations in CIA mice by inhibiting CD31 expression and VEGF. Degenerative changes and inflammatory damage in osteoarthritis were also notably attenuated by chebullinic acid administration. Further studies were performed showing that chebullinic acid greatly inhibited the activation of Erk1/2, p38 MAPK and AKT phosphorylation in human synovial microvascular endothelial cells (HSMECs). This result of this study point to the possible benefit of using chebulinic acid to treat RA targeting angiogenesis (Lu et al., 2020). Resveratrol is a widely existing polyphenol substance, which has pharmacologic benefits such as anti-oxidation, anti-inflammatory and cardiovascular protection, and is widely used in healthcare products and cosmetics (Tian and Liu, 2020; Zhang L. X. et al., 2021). According to Yang et al. research, resveratrol decreased the levels of HIF-1α, MAPK and JNK in IL-1β treated RSC-364 cells. In CIA rats, resveratrol decreased production of diverse pro-inflammatory cytokines, monocyte chemoattractant protein-1 (MCP-1), and reactive oxygen species (ROS). As a result, resveratrol looks to have great potential for clinical validation as an angiogenesis inhibitor (Yang et al., 2018).

#### 4.1.5 Others

Ingredients such as emodin, scopoletin, scopolin and arsenic trioxide can also alleviate RA by inhibiting angiogenesis. Emodin is a natural anthraquinone compound found in a variety of natural medicines such as *Rheum palmatum* L. (Dong et al., 2016). It can dramatically suppress the expression of cytokines, prostaglandin E2 (PGE2), MMP-1, matrix metallopeptidase-13 (MMP-13), VEGF, cyclooxygenase 2 (COX-2), HIF-1a, histone deacetylase 1 (HDAC1) in synovial cells (Ha et al., 2011). In a rat model of AIA, scopoletin inhibits neoangiogenesis and reduces overexpression of VEGF, bFGF and IL-6 in synovial tissue (Pan et al., 2010). Arsenic trioxide (As_2_O_3_) is a highly toxic medicine. It has been clinically used in China as early as 1996 for the treatment of leukemia with remarkable efficacy (Hoonjan et al., 2018). At present, it has been confirmed that the anti-tumor mechanism of As_2_O_3_ is predominantly by inhibiting the proliferation of tumor vascular ECs and inducing their apoptosis, and inhibiting the formation of tumor neovascularization and new lymphatic vessels (Chen J. et al., 2023). Therefore, several studies have used As_2_O_3_ to treat RA (Li C. et al., 2019; Niu et al., 2022). In CIA mice and FLS, As_2_O_3_ dramatically reduced the expression of thrombospondin-1 (TSP-1), transforming growth factor-1 (TGF-1), connective tissue growth factor (CTGF), and VEGF (Zhang et al., 2017). Unfortunately, it has been discovered that As_2_O_3_ has a nephrotoxic effect during leukemia treatment, causing swelling, denaturation, and necrosis of renal tubular ECs. As a result, the use of As_2_O_3_ in the therapy of RA should be approached with caution (Hoonjan et al., 2018).

The content of active components in natural medicines is very low, which greatly limits their development and application. For example, the first-line anti-cancer drug paclitaxel exists in the bark of Taxus brevifolia, which accounts for about 0.004% (Alqahtani et al., 2019). Most of the active substances in natural medicines are directly extracted from plants, but the efficiency of extraction is very low, and a large number of uncontrolled production will lead to the destruction of plant resources (Stanifer et al., 2017; Liu G. Q. et al., 2022).

### 4.2 Natural extracts

Natural medicines treatment of RA has been occupied a very important position since ancient times, including plant extracts and animal extracts. At present, the commercial preparations of natural medicines include tripterygium glycoside tablets, sinomenine hydrochloride enteric-coated tablets, etc., (Zhang Y. et al., 2021; Liu X. et al., 2022). Modern pharmacology has demonstrated that natural medicines extracts have anti-arthritis effects by targeting angiogenesis. Both the total extract and the effective parts had antiangiogenic effect. The natural extracts with anti-arthritis activity were summarized in Table 2.

*Anemone flaccida* F. Schmidt is a perennial plant of the genus Anemone L. in the Ranunculacae family whose roots can be traditionally used as natural medicine. It is mainly rich in triterpenoid saponins, and it has been reported that *A. flaccida* F. Schmidt is effective in the prevention of RA by inhibiting bone differentiation and reconstruction (Kong et al., 2015; Liu et al., 2015). Research by Rao et al. has indicated that *A. flaccida* F. Schmidt effectively suppresses synovial proliferation and angiogenesis in arthritic joints and inhibited VEGFR/PI3K/AKT signaling pathway (Rao et al., 2023).

*Davallia bilabiata* is an essential medicine for the healing of bone injury, which can be used to treat osteoporosis, osteoarthritis (Yang et al., 2014). Liu et al. evaluated the potential anti-angiogenic benefits of *D. bilabiata* through neovascularisation experiments with chicken chorioallantoic membrane (CAM) and migration and tube formation experiments with HUVECs. According to the experimental findings, *D. bilabiata* exhibited anti-angiogenic properties. The anti-angiogenic efficacy of *D. bilabiata* has probably been due to its modulation on matrix metallopeptidase-2 (MMP-2)/tissue inhibitor of metalloproteinase-2 (TIMP-2) balance, suppression of MMP-2 activity and blocking of VEGF ligand/receptors. *Davallia bilabiata* may therefore be a very promising antiangiogenic drug for the management of RA (Liu et al., 2017). *Cissus quadrangularis*, a natural medicine, that has been previously observed to have significant osteoprotective effects (Bafna et al., 2021; Kaur et al., 2021). Administration of *C. quadrangularis* resulted in reduction of serum cytokines, oxidative stress damage and markers of angiogenesis. In addition, the oral LD_50_ of *C. quadrangularis* was over 2000 mg/kg body weight, indicating a high safety profile (Kumar et al., 2015). Saponins from *Nigella glandulifera* seeds, the primary active components, may relieve pain and swelling (Zhao et al., 2013; Zeng L. et al., 2021). In CIA model, rats received saponins10 mg, 50 mg or 250 mg per day for 24 days. The authors found that saponins from *N. glandulifera* seeds modified the immuno-inflammatory response by recovering cytokines, as well as increasing the proportions of Tregs in the peripheral blood vessels and forkhead box protein P3 (Foxp3) levels in knee joints. Saponins from *N. glandulifera* seeds alleviate synovitis, bone degradation, and angiogenesis through the OPG/RANKL/NF-κB and Ang/Tie-2 pathways (Jiang et al., 2022). Total saponins of Rhizoma *Dioscorea nipponica* also have anti-angiogenic effects, the main target of which is to reduce VEGF, Ang-2, and Tie-2 in synovial membrane. In addition to inhibiting angiogenesis by reducing VEGF, total saponins of Rhizoma *D. nipponica* can also inhibit MVD, STAT3 expression, and DNA-binding activity of NF-κB (Liang et al., 2016). The Flavonol-rich Rhus verniciflua Stokes exhibited anti-arthritic potential by decreasing the levels of pro-inflammatory factors, MCP-1 and VEGF in FLS, but downregulated the anti-inflammatory cytokine in CIA mice, resulting in reduced arthritic angiogenesis (Lee et al., 2009). *Dendrobium huoshanense* stem polysaccharide (cDHPS) was able to comprehensively and effectively alleviate arthritis symptoms in CIA mice by inhibiting NF-κB, MAPKs, PI3K/AKT and JAK1/STAT3 signalling pathways. These discoveries indicated that cDHPS has the potential to be utilized in generating of functional foods or medications for the administration of RA (Shang et al., 2021). Moreover, *Spirulina platensis* (Ali et al., 2015), evening primrose oil (El-Sayed et al., 2014) and total Saponins of *Panax japonicus* (Guo et al., 2020) rely on their antiangiogenic effect to prevent the occurrence of arthritis.

Natural extracts can directly or indirectly affect synovial angiogenesis through multiple components and multiple targets. However, there are many components in natural extracts, and the content of effective substances in different batches of plants is different. How to ensure the stable and reliable efficacy of different batches of medicinal materials is difficult. There are still great difficulties in the consistency of natural extracts quality, which seriously hinders the clinical transformation process (Lu et al., 2021).

### 4.3 Prescriptions

There are about 5,000 kinds of natural medicines, and the prescriptions formed by the combination of different natural medicines are countless. Natural medicines has the characteristics of multi-component comprehensive action on multiple targets, which can effectively treat difficult diseases. At the same time, it is praised by more and more people because of its low price and safety (Ziemska et al., 2019; Tang et al., 2020). Following the holism concept of natural medicines, natural medicines prescription offer the benefits of individualized treatment, lower cost and fewer side effects and are proving their worth in managing RA (Liu X. et al., 2022). According to the different types of syndrome of RA in clinical practice, TCM prescription has achieved remarkable clinical effect on RA relief. Classic prescriptions such as Wutou decoction have a history of more than 1,800 years, which is a summary of clinical experience and is still used in RA therapy (Ba et al., 2021; Wu et al., 2021; Xie et al., 2021). With the development of preparation technology, modern preparations have been developed according to the classic prescriptions.

Wutou decoction has been proven in earlier research to have anti-inflammatory and antinociceptive properties. It also seems to prevent the progressive development of chronic arthritic joints and to reverse the symptoms of CIA or AIA rats. The research of He et al. have confirmed that Wutou decoction has remarkable anti-angiogenesis capacities via mediating VEGFR2 and PI3K/AKT/mTOR/HIF-1α signaling pathway (He et al., 2018). Wutou Decoction significantly reduced the generation of angiogenic activators such as VEGFR2, TGF-β, platelet-derived growth factor receptor (PDGF-R) in CIA rats synovium (Ba et al., 2021). Wenluo Yin, a classic prescription composed of a variety of cold-dispersing and pain-relieving herbal medicines. It has been recommended to accelerate blood circulation and so alleviate pain (Li et al., 2002; Guan et al., 2019). Liu et al. evaluated the antiangiogenic efficacy of the Wenluo drink by means of animal tests (CIA rats) and cellular tests (HFLS-RA, HUVEC). The findings indicate that Wenluo drink might significantly lower the density of macrovascular and capillary vessels in synovium. Wenluo Yin inhibits angiogenesis by downregulating various angiogenesis activators (Liu et al., 2013). Qingluo Yin is an empirical prescription for treating dampness-heat arthralgia. It is composed of four kinds of medicinal plant (Li et al., 2002). In CIA rats, Qingluo Yin can suppress angiogenesis, it might achieve so by reestablishing the balance between MMP3 and TIMP-1 in the synovium (Li et al., 2003).

Modern preparations for RA treatment by inhibit angiogenesis include tablets, capsules and patches. Most natural medicines preparations such as Yuxuebi tablet, Kunxian capsule, Shexiang zhuifeng analgesic plaster are used for RA joint swelling, pain and stiffness. YuXueBi tablet decreased disease activity index, bone degradation and angiogenesis in CIA rats. Its anti-angiogenesis mechanism is believed to be the inhibition of CD31 and VEGF production as well as the inhibition of lysyl oxidase (LOX)/Ras/Raf-1 pathway (Su et al., 2022). Shexiang Zhuifeng analgesic plaster significantly decreased the concentrations of cytokines and VEGF in CIA rats, while also lowering the protein overexpression of the AKT/mTOR/HIF-pathway. Shexiang Zhuifeng analgesic plaster decreased nitric oxide (NO) production in RAW 264.7 cells (Wang et al., 2023). In zebrafish embryos, Kunxian Capsule displayed anti-angiogenic actions through the controlling of the PI3K/AKT-MAPK-VEGF pathway (Ma et al., 2023). Sidaxue is a Miao prescriptions known in the research work of Miao medicine. It has been reported that Sidaxue can decrease chronic synovitis and formation of pannus in CIA rats. Network pharmacology investigations suggest that Sidax may primarily impact the PI3K-AKT, TNF-α and NF-κB pathways in RA. Animal studies have confirmed that SX ameliorates inflammatory symptoms, inhibits angiogenesis, downregulates inflammatory factors and regulates the expression of signal transducer and activator of transcription 1 (STAT1), and prostaglandin endoperoxide synthase 2 (PTGS2), resulting in bone protection in CIA rats (Wu et al., 2022). Fengshi Gutong capsule can inhibit the angiogenesis and disease progression of joint synovial tissue in CIA rats, perhaps via inhibiting rheumatoid factor (RF), VEGF, cytokines, intercellular cell adhesion molecule-1 (ICAM1), and Akt (Lin et al., 2021). In addition, Qianghuo Shengshi Decoction (Zeng Z. et al., 2021) and Huatan Tongluo decoction (Chen J. et al., 2019) can also improve RA by inhibiting angiogenesis.

Even though the constitution of the prescriptions of the natural medicines is extremely complex, and the active pharmacological compounds are hard to identify, they are still frequently applied as medicinal products in clinical practice due to their effectivity and the individualization of the dosage. The anti-angiogenic prescriptions are listed in Table 3.

## 5 Conclusions and future directions

Angiogenesis is directly related to a series of inflammatory cell mediators which are part of the overall pathogenic process of RA (Bagli et al., 2004). It has been well established that many kinds of regulating factors are of great relevance in the process of synovial angiogenesis, it is clear that when treating synovial angiogenesis, the inflammatory reaction closely related to it should also be treated. Hence, inhibiting angiogenesis is a very attractive approach for the management of RA by preventing inflammatory infiltration and bone destruction (Maruotti et al., 2006). This paper is a review of the natural medicines components, herbs and prescription that have been found to inhibit angiogenesis in a recent period.

Natural medicines, including bioactives compounds, extracts, and prescriptions have emerged as promising therapeutic alternatives for RA treatment in the modern society. There are many similarities between natural medicines theory of “collaterals” and modern theory of angiogenesis. Most natural medicines with the effect of “clearing collaterals” have better anti-angiogenesis effect (Li and Xu, 2011; Lee et al., 2019; Huang et al., 2020; Jin et al., 2023). It suggests that natural medicines have its own advantages in the intervention of angiogenesis. Tripterygium wilfordii Hook. f. (TwHF), sinomenine, and total glucoside of Paeonia lactiflora Pall. Are probably the most valuable of all the natural products. They are the prescription drug approved by the China Food and Drug Administration for RA treatment. A randomised controlled clinical trial comprising 269 patients compared TwHF with methotrexate (MTX) in the treatment of active RA. TwHF monotherapy was not found to be inferior to, and MTX + TwHF was better than, MTX monotherapy in the control of disease activity in patients with active RA (Lv et al., 2015). In recent years, there have been many investigations of triptolide, which is one of the main chemical components of TwHF. (5R)-5-hydroxytriptolide (LLDT-8) is a new derivative of triptolide with potentially potent immunosuppressive and anti-inflammatory activities which was developed at the Shanghai Institute of Materia Medica. In fact, a phase II clinical trial of this compound has been completed in RA patients (Fan et al., 2018). A open-label, 24-week, parallel randomized controlled trial has shown that the therapeutic effect of methotrexate + sinomenine (MTX + SIN) is similar to methotrexate + leflunomide (MTX + LEF). Notably, gastrointestinal adverse reactions and hepatotoxicity were significantly reduced in patients treated with MTX + SIN compared to patients treated with MTX + LEF (*p* < 0.05) (Huang et al., 2019). Another clinical trial in RA patients showed that SIN treatment markedly improved disease activity and reduced RA-specific clinical indicators such as RF. SIN was found to be effective in remissioning RA, even though it produced a slightly lower remission rate than MTX (Liu W. et al., 2018). Chen et al. demonstrate the hepatoprotective and additive role of total glucoside of Paeonia lactiflora Pall. In combination with MTX and LEF in the therapy of active RA (Chen et al., 2013).

In recent years, the scientific research on natural medicines has made great progress, especially in explaining the material basis, the mechanism, the rationality of the formulation, the quality standard and so on. But, there are still many outstanding problems in the research and development of natural medicines. For example, compared with the pathological process such as synovial inflammation and joint destruction, the study of natural medicines on synovial angiogenesis is relatively insufficient (Cutolo et al., 2022; Komatsu and Takayanagi, 2022; Maeda et al., 2022). The basic research on the efficacy and mechanism of natural medicines is still very weak. Although there have been new developments in the study of angiogenesis models, the models adopted by natural medicines are still relatively simple, and the study on the complex model that can dynamically observe the process of synovial angiogenesis has not been reported (Liu M. et al., 2018). Additionally, most of these studies are currently at the preclinical phase, more specific and adequate clinical research to explore and harness the full potential of natural medicines is sorely lacking.

Since the 1960s, the research on natural medicines has developed rapidly, especially Tu Youyou, who was awarded the Nobel Prize in 2015 for her findings on artemisinin, making the world realize the huge development potential of natural medicines (Tu, 2016). Natural medicines has been applied to the management of tens of thousands of confirmed cases of COVID-19, achieving higher cure rates and lower mortality rates (Low et al., 2023). Natural medicines are an invaluable source for the discovery and development of new drugs. With the ongoing improvement of chemical and biological research techniques, more and more new natural products with novel skeletal structures have been discovered, providing more information on the structure of new drugs and forming a larger library of candidate natural products (Zhang et al., 2020; Chopra and Dhingra, 2021). The comprehensive use of genomics, transcriptomics, proteomics, metabolomics, phenomics and synthetic biology techniques enables the analysis of biosynthetic pathways and key enzymes of complex natural products. Furthermore, the utilization of natural medicines as safe and efficacious drugs could also be promoted by multi-omics technologies and network pharmacology which elucidating the complex mechanism of natural medicines (Chen and Song, 2016; González-López et al., 2021; Pandita et al., 2021). It is also applied in bionic cultivation and synthetic biology of natural products to achieve scientific, standardized and sustainable development of natural products (Clarke and Kitney, 2020; Jamieson et al., 2021; Zhu et al., 2021). Natural medicines are becoming more widely-used due to the rapid advancement of modern preparation technique. The new preparation plays an increasingly crucial role in improving the utilization rate, increasing the solubility and reducing the toxicity of natural medicines (Patra et al., 2018). The defects of natural medicines, such as short half-life in human body and easy to produce accumulated toxicity, can be significantly improved by structural modification using computer aided drug design (Thomford et al., 2018; de Sousa et al., 2022; Mullowney et al., 2023). For example, liu et al. designed and synthesized a series of triptolide-glucose conjugates. These conjugates are well dissolved in water and selectively inhibit the growth of tumour cells, which is worth further exploration (Liu Y. et al., 2022). Therefore, exploring natural products with novel structure and elucidating the mechanism of their anti-angiogenesis will provide more valuable anti-RA lead compounds for the research of innovative drugs. The recognition of lead compounds targeting angiogenesis is an essential contribution to accelerating the early treatment of RA, which may enable the generation of novel therapeutic targets and approaches for the management and prevention of irreversible joint damage.

In conclusion, the efficacy of natural medicines and its natural products in the prevention of RA through anti-angiogenic activity is undoubted. This review elucidates the anti-angiogenic action and mechanism of various prescriptions, herbs and compounds, and provides reference for the application and understanding of natural medicines. It will help to treat RA in its earlier phases, avoiding disability and potentially improving patients’ quality of life.
